# Mass Cytometry in Hematologic Malignancies: Research Highlights and Potential Clinical Applications

**DOI:** 10.3389/fonc.2021.704464

**Published:** 2021-11-05

**Authors:** John M. Astle, Huiya Huang

**Affiliations:** Department of Pathology, Medical College of Wisconsin, Milwaukee, WI, United States

**Keywords:** mass cytometry, cyTOF, hematologic, oncology, acute myeloid leukemia, myeloma, flow cytometry

## Abstract

Recent advances in global gene sequencing technologies and the effect they have had on disease diagnosis, therapy, and research have fueled interest in technologies capable of more broadly profiling not only genes but proteins, metabolites, cells, and almost any other component of biological systems. Mass cytometry is one such technology, which enables simultaneous characterization of over 40 parameters per cell, significantly more than can be achieved by even the most state-of-the-art flow cytometers. This mini-review will focus on how mass cytometry has been utilized to help advance the field of neoplastic hematology. Common themes among published studies include better defining lineage sub-populations, improved characterization of tumor microenvironments, and profiling intracellular signaling across multiple pathways simultaneously in various cell types. Reviewed studies highlight potential applications for disease diagnosis, prognostication, response to therapy, measurable residual disease analysis, and identifying new therapies.

## Introduction and Overview of Mass Cytometry

**Figure 1:**
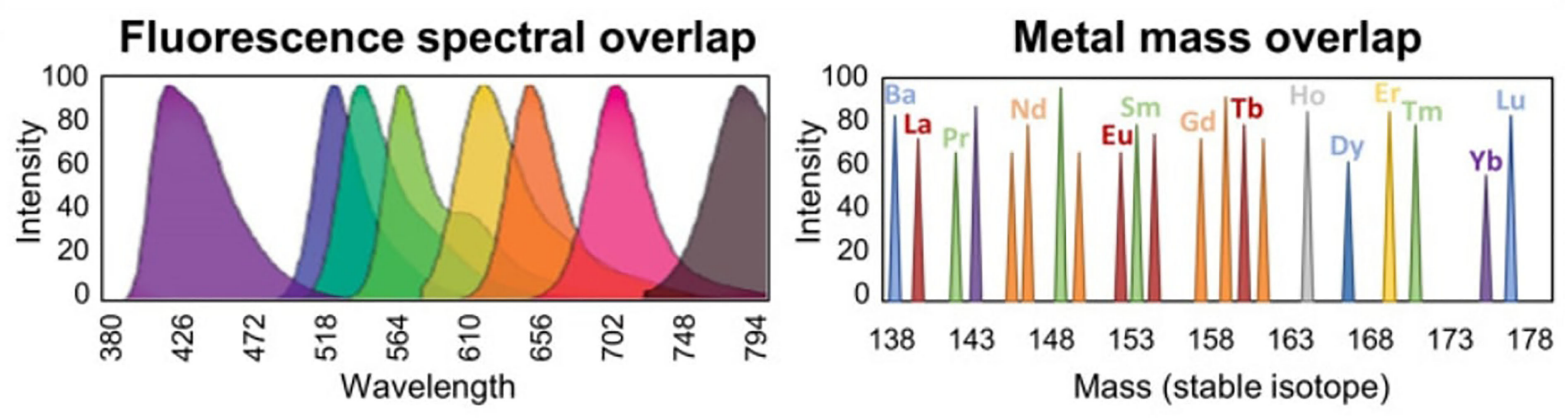
Overcoming spectral overlap *via* mass cytometry. Significant spectral overlap complicates measurement of specific parameters when detecting emitted light from fluorophores in flow cytometry (left). Contamination of adjacent parameters is minimized *via* detection of heavy metal isotopes by mass cytometry (right). Figure adapted from (3).

Mass cytometry, also known as cytometry by time‐of‐flight (CyTOF), combines many aspects of flow cytometry with key advantages of mass spectrometry to enable simultaneous detection of over 40 parameters per cell for up to millions of cells (1, 2). Rather than using antibodies labeled with fluorophores for detection as in flow cytometry, mass cytometry uses antibodies labeled with heavy metals. Detecting the presence and abundance of heavy metals by mass spectrometry minimizes the amount of signal that “spills over” from one parameter to another, significantly decreasing the problem of spectral overlap that plagues flow cytometry (see **Figure 1**). The large number of parameters that can now be reasonably detected on limited specimens enables more thorough characterization, providing a more systemic view of networked processes while at the same time higher resolution of cellular sub-populations and individual cells.

CyTOF was originally developed by Scott Tanner and colleagues at the University of Toronto (1). Simplified, the CyTOF technique begins by incubating fixed cell suspensions and antibodies labeled with heavy metals that are not present in normal biological systems. Rather than individual cells being exposed to a laser and the emitted light detected as in flow cytometry, the cells are nebulized into liquid droplets then vaporized, ionized, and sent through a time-of-flight mass spectrometer, which can determine the identity of each heavy metal based on how long it takes to reach a sensor.

**Figure 2:**
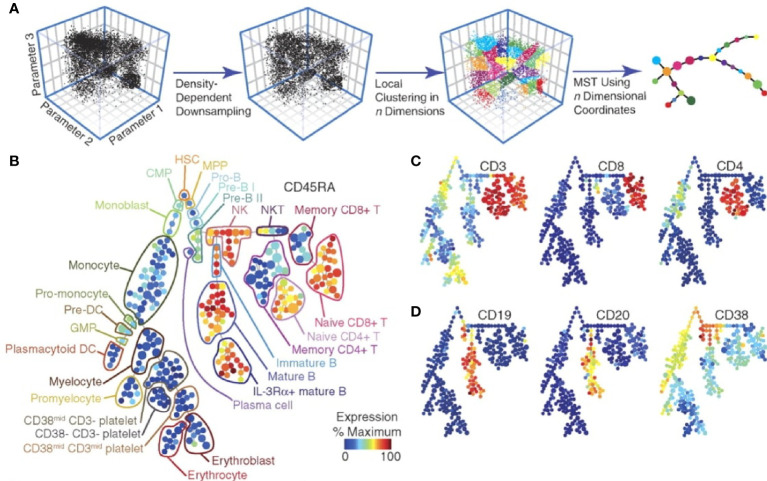
SPADE analysis of multidimensional data. Overview of simplifying multi-dimensional data into a two-dimensional plot by SPADE analysis **(A)**. A tree plot constructed by SPADE software utilizing 13 cell surface markers identified hematopoietic cell populations **(B)**. Expression levels of specific markers can be visualized for each cellular sub-population **(C, D)**. Figure adapted from Bendall et al. (4), with permission.

This new technology first popped onto many people’s radar after Gary Nolan’s group at Stanford (in collaboration with Scott Tanner and others) published a seminal article showcasing the power of this novel technology (4). They stimulated cell populations with various stimuli and simultaneously measured 34 parameters per cell within a population of hematopoietic cells to show differential responses of individual cells and cell sub-populations to these stimuli. By utilizing SPADE analysis software, they were able to visualize an overview of the different cell types and differential expression of various proteins (**Figure 2**). Since that time, there have been continual improvements in technology and reagents, as well as software. Commonly used analysis software include SPADE (5–7), viSNE (8), Citrus (9, 10), and PhenoGraph (11).

This new technology has been utilized to study several different aspects of biology and disease, with many studies thus far emphasizing the characterization of novel subpopulations of various immune cell subsets, characterization of tumor microenvironments, and profiling intracellular signaling across multiple cell subpopulations. Various disease processes have been studied, including COVID-19 (12), Alzheimer disease (13), tuberculosis (14), rheumatoid arthritis (15), non-alcoholic steatohepatitis and hepatocellular carcinoma (16), just to name a few. Potential applications of CyTOF in clinical medicine have been reviewed (17). This mini-review will focus on studies characterizing hematologic malignancies and some of their findings relevant to disease diagnosis, prognostication, response to therapy, measurable residual disease (MRD) analysis, and potential new therapies.

## Research Highlights in Hematologic Malignancies

### Diagnosis

CyTOF has enabled profiling of cellular signaling states, tumor microenvironments, and immune responses to several diseases in a more global fashion than has been done with more traditional approaches, facilitating identification of disease biomarkers. These biomarkers have potential to aid in the diagnosis of various diseases. For example, Han et al. (18) identified disease-specific profiles of intracellular signaling activation that could aid in diagnosis of acute myeloid leukemia. Bailur et al. (19) profiled many markers simultaneously to show that leukemic cells created *via* CRISPR-induced MLL rearrangement were more similar to acute myeloid leukemia (AML) than acute lymphoblastic leukemia (ALL). A similar approach may aid in the distinction between AML and ALL in the subset of cases where this is difficult using currently utilized markers and flow cytometric approaches. Behbehani et al. (20) evaluated cell surface markers in patients with myelodysplastic syndrome (MDS) compared to healthy donors and showed aberrancies in 27 of 31 markers in patients with MDS, the presence of which may be helpful for diagnosing MDS. Van Leeuwen-Kerkhoff et al. (21) showed specific subsets of monocytes are decreased in MDS bone marrows and that these monocytes mediate expansion of a specific T-cell subset, suggesting the identification and quantitation of specific cell subsets may also aid in the diagnosis of MDS. Yang et al. (22) studied tumor microenvironments in follicular lymphoma (FL) and discovered at least 12 subsets of intratumoral CD4(+) T cells, three of which were unique to FL specimens. Subsequent study of FL identified a lack of plasmablasts in FL specimens compared to controls (23). Roussel et al. (24) compared tumor microenvironments of FL, diffuse large B-cell lymphoma (DLBCL) and classic Hodgkin lymphoma (cHL) and identified immune profiles specific to each lymphoma type, including the presence and abundance of monocyte subsets and T-cell subsets. These studies lend evidence to the idea that detecting the presence, absence, or relative abundance of non-neoplastic cell types within a tumor microenvironment can be useful for disease diagnosis.

### Prognostication

CyTOF has also facilitated identification of biomarkers potentially helpful for disease prognostication. Levine et al. (11) published PhenoGraph software for analyzing CyTOF data, which enabled better identification and characterization of leukemic hematopoietic stem and progenitor cells (HSPCs). They showed that cell surface molecules indicating an HSPC immunophenotype are decoupled from intracellular signaling signatures of HSPCs in a significant subset of AML patient samples, and that the number of cells showing intracellular signaling markers of HSPCs correlates more closely with prognosis than when cell surface molecules are used to identify HSPCs. Around the same time Behbehani et al. (25) showed that core binding factor AMLs, which generally have a good prognosis, have an increased fraction of leukemic HSPCs in S-phase of the cell cycle while the relatively poor prognostic FLT3-ITD AMLs have a decreased S-phase fraction. Chretien et al. (26) showed that a “hypomature” NK cell profile or low expression of NKp30 is associated with poor survival in AML patients, while high expression of NKp30 without a hypomature profile is associated with longer survival. Good et al. (27) evaluated 60 primary diagnostic B-lymphoblastic leukemia (BLL) samples to identify 6 features able to predict patient relapse at diagnosis, improving currently established risk stratification methods. Bailur et al. (28) studied tumor microenvironments and identified immune profiles that correlated with disease risk in BLL. Van Leeuwen-Kerkhoff et al. (29) showed thrombomodulin expression on monocytes of MDS patients is associated with lower risk and better leukemia-free survival. Gullaksen et al. (30) profiled intracellular signal transduction in chronic myeloid leukemia (CML) and clear differences were noted between CML cells and healthy donor cells. Changes in signaling were detected within three hours of nilotinib therapy, and profiles identified after seven days of therapy correlated with BCR-ABL1 burden at 3 and 6 months, suggesting early signal transduction profiles may be useful for prognostication. Yang et al. (22) showed that naïve T cells within the tumor microenvironment of FL were associated with improved survival, while specific subsets of PD-1(+) T cells and loss of the costimulatory receptor CD27 on intratumoral T cells were associated with poor survival. Yang et al. (31) then showed that T-cell immunoglobulin and ITIM domain (TIGIT) is highly expressed on exhausted intratumoral T cells of FL and is associated with inferior survival. These studies show that the types of biomarkers identifiable by CyTOF for disease diagnosis such as intracellular signaling profiles and cellular profiles within tumor microenvironments may be helpful for disease prognostication.

### Response to Therapy and Vaccination

CyTOF has been used to better characterize responses to various therapies, leading to better understanding of mechanisms of action as well as mechanisms of resistance to therapy. Saenz et al. (32) used CyTOF to help evaluate the response of post-myeloproliferative neoplasm secondary AML (sAML) cells to different therapies in a xenograft model, facilitating a broad and detailed characterization of drug responses, suggesting BET protein proteolysis-targeting chimera may be more effective than bromodomain inhibitor in treating this neoplasm. This group also showed decreased expression of Bcl-xL, CDK4/6, c-MYC, IL-7R, p-STAT5, and PIM1, and increased expression of BIM, HEXIM1, and p21 in BET inhibitor treated AML cells from sAML. Synergistic activity of BET inhibitor with the JAK inhibitor ruxolitinib is seen in sAML cells, and there is also synergistic activity of BET inhibitor and HSP90 inhibitor against ruxolitinib-resistant sAML cells. Zeng et al. (33) evaluated 24 primary AML samples to perform detailed characterization of mTOR inhibition in AML cells, and results pointed to cell signaling pathways dampening drug response. Edwards et al. (28) characterized CSF1R signaling to show anti-leukemic activity of CSF1R inhibitors occurs through inhibiting paracrine signaling from support cells. In 2020 Han et al. (34) showed that cobimetinib antagonized pERK and pS6 signaling, and was associated with increased BCL2 expression in leukemia HSPCs in venetoclax-sensitive AML specimens. Borthakur et al. (35) published findings of a phase-I study of combined sorafenib, plerixafor, and G-CSF for AML, where CyTOF was used to identify resistant sub-clones and characterized signaling within these cells to show persistent Akt and/or ERK signaling. Rorby et al. (36) investigated the mechanism of synergistic activity of midostaurin in combination with daunorubicin and cytarabine, which has been shown to increase survival in FLT3-mutated AML patients. They showed that cytarabine appeared to antagonize midostaurin’s effect on protein phosphorylation and increased surface expression of FLT3. Deng et al. (37) helped identify VEGFR2 signaling as the mechanism by which Apatinib exerts its anti-leukemic effect in BLL. Bandyopadhyay et al. (38) showed that avasimibe, an inhibitor of cholesterol esterification, synergistically suppressed CML cell proliferation, and led to downregulation of the MAPK signaling pathway, which likely sensitized CML cells to imatinib. Baughn et al. (39) identified loss of CD56 and CD66a and a signature of activation are associated with proteasome inhibitor resistance in a myeloma cell line suggesting these have potential as markers of resistance to proteasome inhibitor therapy. Adams et al. (40) characterized the myeloma tumor microenvironment and showed that daratumumab-treated patients have increased cytotoxic T cells and reduced immunosuppressive cell populations, consistent with immune modulation as a novel mechanism of action for daratumumab. Teh et al. (41) characterized regulators of cell death, mitosis, cell signaling, and cancer-related pathways in myeloma cells treated with dexamethasone or bortezomib to identify increased CREB and MCL-1 in treatment-resistant myeloma cells. Visram et al. (42) evaluated bone marrow from 13 newly diagnosed, 11 relapsed pre-daratumumab, and 13 triple-refractory myeloma patients and found that patients who are resistant to three lines of therapy have a distinct immune tumor microenvironment including decreased CD4(+) T cells and naïve T cells compared to newly diagnosed and relapsed but pre-daratumumab treated patients. This finding suggests that immune microenvironment signatures may help predict response to therapy. Finally, Alimam et al. (43) monitored immune responses to influenza A vaccine in 7 polycythemia vera (PV), 8 essential thrombocythemia (ET), and 4 myelofibrosis (MF) patients compared to 6 healthy donors, and evidence of impaired B- and T-memory cell responses in patients with myeloproliferative neoplasms was identified. The ability of CyTOF to broadly characterize numerous antigens and cells simultaneously greatly facilitates evaluation of responses to various therapies and even vaccinations. Several of these studies also highlight the ability of CyTOF data to elucidate the mechanism of action of drugs, and to identify potential predictors of response or resistance to therapy.

### MRD Analysis

MRD analysis is typically performed using one or a combination of two distinct approaches. The first approach is to identify populations of cells that are immunophenotypically similar to the immunophenotype at diagnosis. This is the easier of the two approaches in many respects, but falls short when tumors modulate their immunophenotype, which some neoplasms do quite readily. The second approach is to identify immunophenotypes that are different from the immunophenotype of a normal cell counterpart to the tumor cell. For example, a “different-from-normal” approach to MRD analysis for AML would look for populations of aberrant HSPCs that display a different immunophenotype than normal HSPCs. A study by Ferrell et al. (44) highlighted the power of CyTOF for characterizing how versatile AML cells can be at modulating their immunophenotype. They characterized 46 samples from 5 AML patients before, during, and immediately after induction chemotherapy to show how the AML population and subpopulations modulated their immunophenotypes over time. Studies such as this not only characterize the degree of versatility with which cancers can modulate their immunophenotype, but may help identify new cancer-specific immunophenotypes and their frequency in studied populations. Unfortunately, the rate at which cells flow through CyTOF machines is roughly an order of magnitude slower than flow cytometers frequently used in clinical laboratories, limiting its utility for clinical use. However, CyTOF can facilitate the discovery of disease-specific immunophenotypes including specific combinations of immunophenotypes much more readily than other approaches due to the sheer number of antigens that can be monitored from a given, often limited, specimen. Despite the limited number of specimens that could be run in a day, a mass cytometer may be warranted for MRD detection in patients where MRD analysis has been hindered by the use of new antibody therapeutics. For example, antibodies targeting B-cell antigens are being used to treat B-cell lineage leukemias and lymphomas, and the antigens that these antibodies target (which are usually the same antigens used to identify the presence of these cell populations by flow cytometry) are often rendered useless as markers for MRD detection. The ability to identify cancer cell populations using combinations of other antigens by CyTOF could be extremely useful. Even if CyTOF machines are not used in clinical laboratories in the near future, software developed for CyTOF may prove useful for MRD detection. Amir et al. (8) showed that viSNE software can be used to visually distinguish leukemia from healthy bone marrow samples, and the potential of viSNE for identification of MRD was shown. Similarly, Bandyopadhyay et al. (45) utilized CyTOF to identify a subpopulation of cells in secondary AML that was not readily identified by manual gating approaches of two-dimensional plots, highlighting the power of software facilitating evaluation of multiple dimensions at once. As technological improvements increase acquisition rates, costs decrease, and data validating the value of high-parameter approaches to MRD analysis are published, clinical laboratories may in the not-too-distant future begin using CyTOF for MRD analysis.

### Potential New Therapies

New discoveries identified *via* CyTOF analysis can point to potential new therapies, and potential new therapies can be tested in model cells and organisms to characterize their effects and side-effects across multiple signaling pathways in multiple cell types at once. Han et al. (18) identified distinct patterns of signaling activation within leukemia stem cells across AML samples and between AML and control samples. mTOR signaling was identified as a possible therapeutic target as mTOR regulated proteins 4EBP1 and S6 were found to be phosphorylated in FLT3-ITD progenitor cells but not in controls. This study highlights the importance of using high dimensional cytometry to monitor multiple signaling pathways simultaneously to identify possible therapeutic targets. Zeng et al. (33), after identifying cell signaling pathways that dampen drug response during mTOR inhibitor administration, showed that targeting these pathways along with mTOR inhibition led to increased efficacy. Sarno et al. (46) set out to study why high expression of CRLF2 is associated with poor prognosis in patients with B-lymphoblastic leukemia/lymphoma, and identified coordinated signaling involving JAK/STAT, PI3K and CREB pathways downstream of CRLF2. The authors showed in primary leukemia cells that SRC/ABL inhibition was more effective at inhibiting the CRLF2-driven network than JAK or PI3K inhibition. Fisher et al. (47) identified increased activity of JAK-STAT, MAPK, PI3K and NFkB signaling in myelofibrosis (MF) and sAML cells. They identified constitutive and hypersensitive NFkB signaling in MF and sAML HSPCs, and found that inhibition of NFkB in CD34(+) HSPCs from MF patients suppressed myeloid colony formation, suggesting NFkB inhibition as a potential therapeutic approach in MF and sAML. They then monitored cytokine production in MF patient blood cells by CyTOF, and monocytes were found to overexpress many cytokines induced by thrombopoietin, TLR ligands, and tumor necrosis factor (48). All of these cytokines could be suppressed by inhibiting NFkB and/or MAPK signaling, suggesting this as a novel pharmacotherapy in MF. Teh et al. (41), after identifying increased CREB and MCL-1 in treatment-resistant myeloma cells, showed that combining an MCL-1 inhibitor to dexamethasone showed synergistic activity in killing primary myeloma cells from patients. These are just a few examples of how high-parameter profiling *via* CyTOF can help identify new potential therapies for diseases.

## Discussion

Mass cytometry has significantly increased our capacity to profile entire populations of cells at the individual cellular level. This can be extremely useful, particularly when the total number of cells for evaluation is limited, such as in clinical biopsy or fine needle aspirate specimens. However, it is important to recognize that CyTOF is not completely equivalent to flow cytometry with more measurable parameters. Slower acquisition rate, more complicated data analysis, and need for surrogates of forward and side scatter are a few of the caveats to the promise of mass cytometry for clinical use.

Review of the current literature has shown that only a few hematopoietic malignancies such as AML and plasma cell myeloma have been investigated *via* CyTOF in more than a handful of studies. Numerous diseases not mentioned in this mini-review could benefit from more detailed characterization made possible by this new technology. A major strength of CyTOF is in its ability to identify not only individual biomarkers but combinations of markers that together provide more systemic or global signatures of diseases or biological states (similar to gene expression profiling). These signatures may incorporate relative abundance of different cell subsets, expression levels of different proteins, and/or activation states of various cellular signaling pathways. Such expression profiles may be useful for disease diagnosis, prognostication, and predicting response to therapy. While the relatively slow acquisition rate of current CyTOF machines limits their utility for clinical MRD analysis, CyTOF provides a powerful mechanism for identifying disease-specific markers (i.e. leukemia-specific immunophenotypes) that can be used in routine flow cytometric MRD analysis. CyTOF has been particularly useful for characterization of tumor microenvironments. Continued research utilizing CyTOF will undoubtedly promote efforts to move this powerful technology into the clinic.

## Author Contributions

HH and JA contributed to the concept development, review of literature and writing of manuscript. All authors contributed to the article and approved the submitted version.

## Conflict of Interest

The authors declare that the research was conducted in the absence of any commercial or financial relationships that could be construed as a potential conflict of interest.

## Publisher’s Note

All claims expressed in this article are solely those of the authors and do not necessarily represent those of their affiliated organizations, or those of the publisher, the editors and the reviewers. Any product that may be evaluated in this article, or claim that may be made by its manufacturer, is not guaranteed or endorsed by the publisher.
